# Optimization of Osmotic Dehydration of Sapodilla (*Achras zapota* L.)

**DOI:** 10.3390/foods11060794

**Published:** 2022-03-10

**Authors:** Lívia Muritiba Pereira de Lima Coimbra, Adrielle Zagmignan, Paulo Victor Vieira Gomes, Jânaira Farias Araujo, Gabrielle Damasceno Costa dos Santos, Rita de Cássia Mendonça de Miranda, Silvana Magalhães Salgado, Samara Alvachian Cardoso Andrade, Luís Cláudio Nascimento da Silva

**Affiliations:** 1 Departamento de Nutrição, Universidade CEUMA, São Luís 65075-120, MA, Brazil; adrielle.zagmignan@ceuma.br (A.Z.); paulo106983@ceuma.com.br (P.V.V.G.); araujo.janairafarias@gmail.com (J.F.A.); 2 Laboratório de Patogenicidade Microbiana, Universidade CEUMA, São Luís 65075-120, MA, Brazil; gabrielledamasceno.nutri@gmail.com (G.D.C.d.S.); luiscn.silva@ceuma.br (L.C.N.d.S.); 3 Rede de Biodiversidade e Biotecnologia da Amazônia Legal (BIONORTE), Universidade Estadual do Maranhão, São Luís 65055-310, MA, Brazil; rita.miranda@ceuma.br; 4 Laboratório de Microbiologia Ambiental, Universidade CEUMA, São Luís 65075-120, MA, Brazil; 5 Departamento de Nutrição, Universidade Federal de Pernambuco, Recife 50670-901, PE, Brazil; silvana.salgado@ufpe.br; 6 Departamento de Engenharia Química, Universidade Federal de Pernambuco, Recife 50670-901, PE, Brazil; samaraandrade@uol.com.br

**Keywords:** preservation of food, sapoti, sensory analysis, microscopy, loss of water

## Abstract

Sapodilla (*Achras zapota* L.) is a fruit with a great nutritional potential; however, its perishable nature is a great obstacle for commercialization/exportation. Herein, osmotic dehydration was applied to sapodilla to reduce post-harvest losses and obtain a stable product with acceptable sensorial characteristics. Initially, a 2³ full-factorial design was performed to determine the effect of temperature (30–50 °C), sucrose concentration (40–60% °Brix) and immersion time (90–240 min) on the moisture loss (ML), solid gain (SG) and dehydration efficiency index (DEI). The samples with higher DEI values were subjected to sensory analysis, followed by physicochemical, microbiological and structural analyses. The temperature and the concentration of the osmotic solution had significant influence (*p* < 0.05) on ML and SG, whereas DEI was significantly influenced (*p* < 0.05) by the concentration of osmotic solution and the immersion time. The sample produced by osmotic dehydration using the optimized conditions (40 °C, 50 °Brix; 165 min) obtained higher scores on the sensorial attributes, greater compliance with microbiological standards and generated turgor reduction and ruptures of sapodilla cell walls.

## 1. Introduction

*Achras zapota* L. (synonym of *Manilkara zapota* (L.) P.Royen) is a medium-sized evergreen species from the Sapotaceae family native to tropical areas of the Americas (from southern Mexico to parts of South America) and now also cultivated in other tropical regions throughout the world [1,2]. In Brazil, the tree is known as ‘Sapotizeiro’ and has good adaptation in the northeast region of Brazil due to appropriate soil and climate conditions [3,4]. The fruit is named as ‘sapoti’ in Brazil, and it is also known as ‘chicozapote’ or ‘sapodilla’ in other Latin countries. It has ovoid shape, brown bark and a soft pulp with sweet and slightly astringent taste [5]. The aroma of fresh fruit is typically minty, fatty/green and woody, containing at least 13 aroma-active compounds [6]. This fruit has been applied for the production of juices, wine, ice-creams, jam, cookies and other food formulations [5,7,8,9].

The fruits are sources of carbohydrates, pro-vitamins A, vitamin C and B complexes and minerals such as calcium, phosphorus and iron. These fruits have an average of 72 to 78% humidity and a high percentage of post-harvest losses [3,10,11]. Although sapodilla has a great nutritional potential, its perishable nature is a great obstacle for commercialization/exportation: in natural conditions, it can be stored for up to 15 days [12]. In this sense, few studies have been performed in order to extend the shelf-life of this fruit [13,14,15,16,17,18].

The industrial processes aiming to reduce the water activity appear as an alternative to solve and/or minimize the problems of post-harvest losses of sapodilla and, consequently, to facilitate its commercialization. In this context, osmotic dehydration is an alternative to reduce the initial moisture of the fruit by an average of 20% to 50% [19]. Osmotic dehydration has mainly been used as a pre-treatment for some conventional processes, such as air drying. The process of osmotic dehydration consists of the immersion of food in a concentrated solution, which results in the water leaching from the fruit into the solution, the migration of solutes from the solution to the fruit and the leaching of natural solutes of the food fabric (sugars, organic acids, minerals and vitamins) to the osmotic solution. The latter is not significant in quantitative terms [3,20,21,22].

Some advantages of osmotic dehydration over other drying processes can be cited, such as the greater preservation of natural color and flavor, the inhibition of enzymatic browning during subsequent drying, low energy consumption and improved final product quality [23]. The most commonly used osmotic agents are glucose, sodium chloride, sucrose, glycerol, sorbitol, corn syrup, glucose syrup and fructooligosaccharides. These must be carefully selected for different foods; for example, sodium chloride is used for processing vegetables, whereas sucrose has commonly been used as an osmotic agent for fruits [24].

Sucrose is considered one of the best dehydrating agents, especially when dehydration is used as a pre-treatment for drying. In addition, the use of sucrose in fruits generates products with similar sensory characteristics between natural and dry products. In this study, sucrose was considered due to its ease of access (physical and financial) which facilitates the reproducibility of the process. In summary, sucrose is a conventional and accessible osmotic solution, from a physical and financial point of view, and has efficiently been applied for a range of products [25,26,27,28].

In view of the above, this research had the objective of obtaining osmotically dehydrated sapodilla, with a maximum loss of water and minimum gain of solutes, with good organoleptic characteristics, microbiological and structural stability.

## 2. Materials and Methods

### 2.1. Material

The sapodilla fruits (*Achras zapota* L.) were randomly purchased from a local supermarket in Recife, PE, Brazil. The fruits were visually selected by color (completely brown), size (mean diameter 4.6 cm), ovoid shape and an absence of physical damage. Commercial sucrose was used as the osmotic agent.

### 2.2. Osmotic Dehydration

The general aspects of raw material were visually inspected, and the samples were weighted and selected according to the degree of maturation (12 to 16 °Brix). Afterwards, the material was washed in running water and sanitized using a sodium hypochlorite solution (2.5% *w/v*) for 15 min. Subsequently, the fruits were manually peeled with a stainless-steel knife. The fruits were cut into longitudinal slices (5 × 1 × 1 cm) and the seeds were removed. The samples were subjected to a bleaching process using flowing steam (100 °C for 5 min in each side) and then immediately cooled in ice water for 5 min. After bleaching, the slices were drained on paper towels, weighed and immersed in the sucrose solution (sample/solution ratio of 1:20) at 110 rpm in a shaker (Marconi, MA-410) under predetermined temperature, time and concentration (Table 1). The excess osmotic solution was removed by absorbent paper. The samples were weighed again and dried at 60 °C (±5 °C) in an oven with air circulation. After 4 h, the dehydrated fruits were bagged in low-density polyethylene bags and stored at room temperature.

### 2.3. Experimental Design

A 2^3^ factorial design was carried out, with 8 factorial points (levels ±1), 3 central points (level 0) and 6 axial points (±α), totaling 17 assays. The independent variables were temperature (T; 30 °C to 50 °C), concentration of osmotic solution (C; 40 °Brix to 60 °Brix) and immersion time (t; 90 min to 240 min). The dependent variables were moisture loss (ML), solid gain (SG) and the Dehydration Efficiency Index (DEI = ML/SG).

The obtained data were adjusted to the following polynomial:

(1) $$Y=\phi (T,C,t)=\beta_{0}+\beta_{1}T+\beta_{2}C+\beta_{3}t+\beta_{11}T^{2}+\beta_{22}C^{2}+\beta_{33}t^{2}+\beta_{12}TC+\beta_{13}Tt+\beta_{23}Ct$$

where *βn* are the regression coefficients, *Y* is the response in question (ML, SG and ML/SG) and *T*, *C* and *t* are the independent variables (temperature, concentration of osmotic solution and immersion time, respectively). The responses of interest were thus defined:

-Moisture Loss (ML)—calculated in percentage terms, based on the initial weight of the material:

(2) $$\mathrm{ML}(\%)=\frac{\left(\mathrm{UixMi}-\mathrm{UfxMf}\right)}{\mathrm{Mi}}$$

where ML(%) is the loss of moisture in relation to initial material, Mi= is the initial moisture content of matter (%), Uf is the final moisture content of matter (%), Mi is the total initial mass of matter (%), and Mf is the total final mass of matter.

-Solid Gain (SG)—calculated through a balance of mass acquired during the processing of solids:

(3) $$\mathrm{SG}(\%)=\frac{\left({}^{\circ}\mathrm{BrixfxMf}-{}^{\circ}\mathrm{BrixixMi}\right)}{\mathrm{Mi}}$$

where SG (%) is the solid gain, °Brixi is the initial total soluble solids content of the material, °Brixf is the final content of total soluble solids of the material.

-Dehydration efficiency index (DEI): ML/SG

The experimental design was built, and the data were analyzed by a Student’s *t*-test through the statistical software Statistica 6.0 at the 5% level of significance using the StatSoft Inc., Statistica: Data Analysis Software System, Version 7 (2004).

### 2.4. Analytical Methods

#### 2.4.1. Sensory Analysis

The assays with higher Dehydration Efficiency Indices (DEIs) were subjected to the acceptance analysis, using a structured nine-point hedonic scale (1 = very disagreeable, 9 = very much liked) to evaluate the attributes: aroma, color, flavor, texture and quality. The intention of purchase was evaluated by applying a 5-point hedonic scale which varied from 1 = “I would certainly not buy” to 5 = “I would certainly buy”. Fifty tasters were randomly recruited among students and professors of the ‘Departamento de Ciências Domésticas’ (DCD/UFRPE; Recife, PE, Brazil), with the exclusion criterion of an aversion to sapodilla. Samples were randomly coded with three-digit numbers. The acceptability index (AI) for the overall quality attribute was calculated: AI(%) = Y × 100/Z (Y = average score obtained for the product; Z = maximum obtained score). This study was approved by the Research Ethics Committee, through the Brazil platform (Process: 331.686). The in natura *Sapodilla* and the sample that showed the best results in the sensory analysis were submitted to the analyses described in Section 2.4.2, Section 2.4.3 and Section 2.4.4. All analyses were performed in triplicate.

#### 2.4.2. Centesimal Composition and Physicochemical Parameters

The following analyses were performed before and after osmotic dehydration of sapodilla: soluble solids by direct reading in an ATAGO manual refractometer expressed in °Brix; weight of the samples in a semi-analytical balance of the Tecnal brand; humidity, lipids, ashes, proteins, dietary fiber and pH (AOAC, 2002); carbohydrates [29]; total caloric value (TCV) by the calculation method; and water activity (Aw) in Decagon Aqualab 4 TE equipment.

#### 2.4.3. Microbiological Analysis

The quantification of *Salmonella* sp. (CFU/25 g; method 996.08) and total coliforms at 45 °C (MPN/mL; method 991.14) was performed in triplicate [28,30].

#### 2.4.4. Structural Analysis

The images were generated with a Scanning Electron Microscope (SEM; Quanta 200 FEG). In brief, the samples were fixed using 2.5% glutaraldehyde and in 0.1 M cacodylate buffer. Subsequently, the samples were washed in 0.1 M cacodylate buffer (three times). The tissue was post-fixed in 2% osmium tetroxide and 0.1 M cacodylate buffer. The dehydration process occurred using a graded acetone series (30%, 50%, 70%, 90% and three times at 100%; each procedure lasted 10 min). At the end of this stage, the samples were dried for 1 h and 10 min at 40 °C and 80 bar. The samples were then mounted on the stubs and sprayed with gold for 80 s. After this procedure, the samples were visualized in the SEM.

### 2.5. Analysis Statistics

All the experiments were carried out in triplicate and the results are expressed as mean values. A *p*-value < 0.05 was considered statistically significant. The data were analyzed using the statistical software Statistica 6.0.

## 3. Results and Discussion

In this study, a 2^3^ factorial design approach was employed in order to optimize the process of osmotic dehydration of sapodilla. It was observed that the moisture loss (ML) was higher than the solid gain (SG) in all 17 assays (Table 2). The differences between ML and SG are due to the larger size of sucrose molecules relative to water molecules, which allows water molecules to move faster than sucrose molecules [25]. Similar results were found when the osmotic dehydration was applied for apricot (*Prunus armeniaca* L.) [31], apple cv. “Idared” [32] and acerola (*Malpighia punicifolia*) [22]. These results confirmed the main aim of osmotic dehydration, which is to achieve maximum water loss with minimum solid gain.

A regression analysis was applied to model the ML, SG and DEI values as quadratic functions of the osmotic solution concentration, immersion time and temperature. The regression coefficients for the obtained models are presented in Table 3. The statistical analysis indicated that the adjusted models were considered predictive, not having a significant lack of adjustment and with R^2^ values very satisfactory for all the answers. The R^2^ values for ML, SG and DEI were 0.982, 0.979 and 0.947, respectively. Similar results were found when studying the mass transfer kinetics of radish cylinders (R^2^ > 0.97) [33] and banana cylinders (R^2^ > 0.98 for ML and SG) [26].

**Figure 1:**
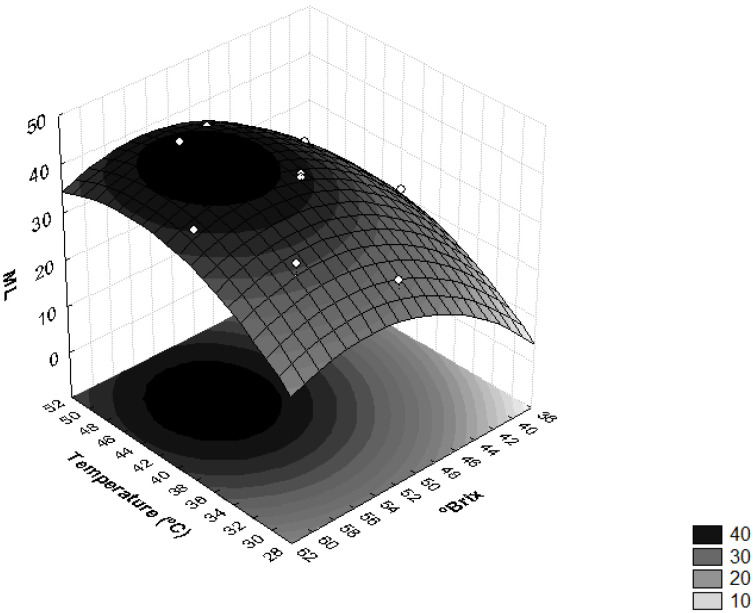
Surface response for moisture loss (ML) as a function of the concentration of sucrose solution (°Brix) and temperature (°C).

Figure 1 shows the influence of sucrose concentration and temperature on moisture loss. This verified that the interaction between the concentration of the osmotic solution and temperature induced significant effects on the ML levels. Higher moisture losses were obtained when the osmotic solution had concentrations higher than 50 °Brix and the operating temperatures were higher than 40 °C.

These results can be explained by the increase in the osmotic pressure gradient, as well as by the use of high temperatures which cause a decrease in the viscosity of the osmotic medium and lead to swelling, plasticization and destruction of the cell membrane structure, i.e., increased permeability of the membrane, which, in turn, favors water loss [22,26]. Similar results were obtained with the osmotic dehydration of apricots [31], where the use of higher concentrations of sucrose led to higher osmotic pressure gradients, resulting in higher solid gains and water loss over of the osmotic treatment period [21].

Sucrose solution was applied in the osmotic dehydration of fresh *Terung Asam* (*Solanum lasiocarpum* Dunal), and verified that the increase in sucrose concentration and immersion time accelerated ML. The authors discussed that these results are due to the osmotic gradient between the hypertonic sucrose solution and the intracellular fluid of the *T. Asam* slices [27]. This gradient promoted the diffusion of water from the samples to the osmotic medium. Therefore, accelerated water removal can be achieved by increasing the solution concentration to create a higher osmotic driving force. Similar results were found in the osmotic dehydration of ripe papaya (*Carica papaya* L.) with various sucrose concentrations (40, 50 and 60 °Brix), where greater ML and SG were archived at the highest sucrose concentrations [34]. Regarding temperature, similar results were achieved in the osmotic dehydration of banana (*Musa sapientum* shum.) at temperatures from 25 to 55 °C, salt concentrations of 0–10 g/100 g and sucrose concentrations of 30–60 g/100 g [26].

**Figure 2:**
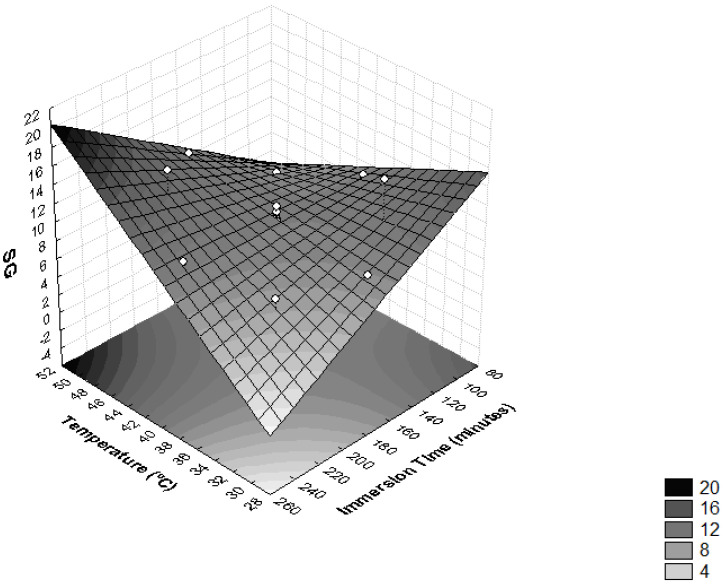
Surface response for solid gain (SG) as a function of immersion time (minutes) and temperature (°C).

The interactions between temperature and time of immersion on SG is shown in Figure 2 and Table 3. It was observed that these variables had significant influences on SG (*p* < 0.05). The highest levels of SG were obtained when the operating temperature was higher than 46 °C and immersion time was more than 210 min. Higher SG values were also achieved when temperatures lower than 34 °C were combined with immersion times under 120 min. Similar results were found in the process of the dehydration of apple cylinders using four different osmotic media (50% sucrose, 49.47% sucrose + 0.25% CaCl_2_, 48.27% sucrose + 0.5% CaCl_2_ and 40.52% sucrose + 0.50% CaCl_2_ + 2% NaCl) at temperatures of 20, 40 and 60 °C at 30, 60, 120 and 180 min intervals [35]. During the dehydration of *T. Asam*, it was also found that the SG was accelerated by the increase in temperature and immersion time, with the maximum SG recorded at a temperature of 55 °C and immersion time of 210 min [27].

The impact of temperature on moisture loss kinetics without any effect on solids gain is most obvious between 30 °C and 60 °C for vegetables and fruits [36]. It is reported that temperatures above 60 °C should be avoided because they reduce the quality of the final product, altering the structure of the cell membranes, resulting in a loss of selectivity, leading to greater incorporation of solutes in the fruit. Furthermore, elevated temperatures may induce significant changes in the texture and nutritional composition of the food as a consequence of nutrient losses from the product to the osmotic solution [26].

**Figure 3:**
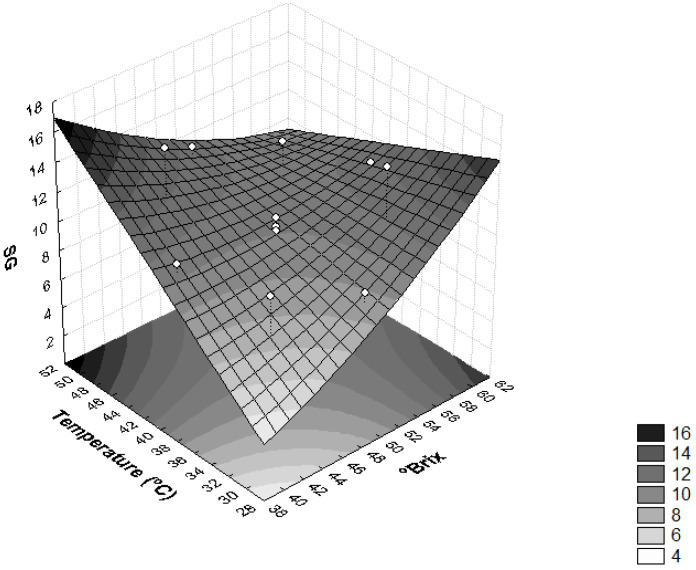
Surface response for solid gain (SG) as a function of the concentration of sucrose solution (°Brix) and temperature (°C).

The influence of temperature and concentration of the osmotic solution on SG was also found as significant (*p* < 0.05) (Figure 3). Higher temperature values (T > 46 °C) and lower solution concentration values (<44 °Brix) provided higher SG, whereas temperatures <34 °C and concentrations of solution of sucrose <44 °Brix resulted in a product with lower SG, thus leaving the final product closer to in natura conditions. The same behavior observed for sapodilla was observed for dehydrated radishes and pumpkins [37], i.e., the temperature and concentration of the osmotic solution influenced SG significantly (*p* < 0.05).

High SG values in the osmotic dehydration process are undesirable because they can lead to sensory changes in the product. Furthermore, when the concentration of solutes in plant tissues is high, the efficiency of dehydration is reduced due to the formation of an external barrier that hinders mass transfer [28].

**Figure 4:**
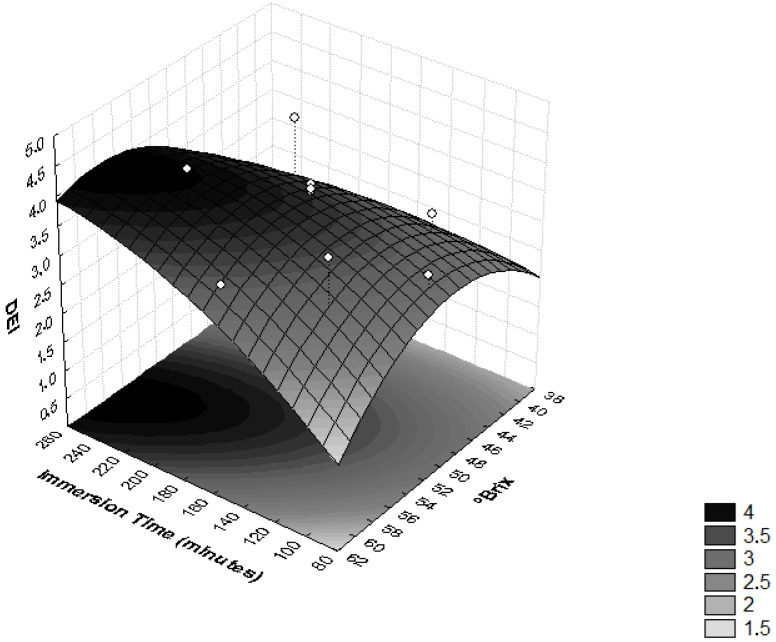
Surface response for the Dehydration Efficiency Index (DEI) as a function of immersion time (minutes) and the concentration of sucrose solution (°Brix).

In order to evaluate the influence of osmotic dehydration parameters on the ML and SG efficiency, the DEI values were determined. In Figure 4, it is possible to observe that the interaction between the sucrose osmotic solution concentration and the immersion time significantly influenced the DEI (*p* < 0.05). High values of this relationship are related to good dehydration conditions [37], because they reflect a treatment that provides high moisture loss and low solute absorption [28].

An increase in the concentration of the osmotic solution implies high osmotic pressure and, consequently, higher mass transfer rates [38]. Osmotic duration is another important variable that has an unavoidable influence on the mass transfer rate. It was noted that the prolonged immersion time improved the ML [27]. During the dehydrating cylinders of pumpkins, it was observed that the DEI increased with higher immersion time, temperature and, especially, concentration of the sucrose solution [37].

**Figure 5:**
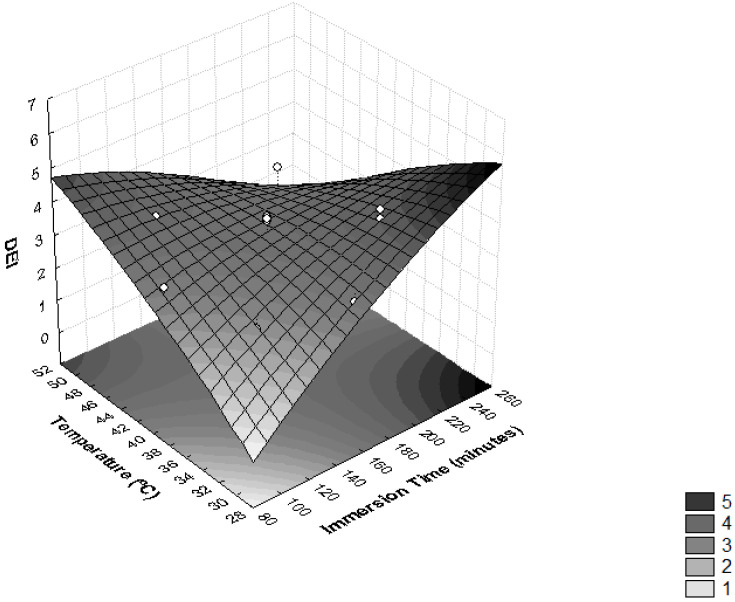
Surface response for Dehydration Efficiency Index (DEI) as a function of immersion time (minutes) and temperature (°C).

As presented in Figure 5, higher values of DEI were reached with values of temperature >48 °C and immersion times <120 min, and temperatures <40 °C and immersion times >165 min. These effects were observed in assays 7, 5 and 9, where the highest values of DEI were obtained (Table 2). Similar results were seen in the osmotic dehydration process of *T. Asam* slices, where the optimal process conditions were predicted as a temperature of 38.1 °C, sucrose concentration of 55.6% and osmotic duration of 126.3 min [27].

Given the results, assays 7 and 9 obtained satisfactory ML and were chosen to carry out the sensory analysis (Table 2). Taking this criterion into account, assay 5 was eliminated.

### 3.1. Sensory Analysis

The means of acceptance scores are presented in Table 4. Assays 7 (34 °C, 56 °Brix, 210 min) and 9 (40 °C, 50 °Brix, 165 min) did not differ statistically (*p* < 0.05) among the evaluated attributes, although assay 9 obtained higher averages (Table 4). In relation to the intention to buy, it was found that assay 9 also obtained a higher percentage of “certainly buy” (32%), whereas less individuals chose this for assay 7 (22%) (Table 5). Regarding the acceptability index for the overall quality attribute, similar results were found for both samples tested: 71.55% (assay 7) and 75.11% (assay 9). These values (>70%) are considered as sensorially acceptable products [39]. The different conditions of the osmotic dehydration process had a notable influence on the acceptability of the final product. In view of the results of the acceptability index and purchase intention, assay 9 was selected for microbiological, physicochemical and structural analyses.

### 3.2. Microbiological Analysis

The dehydrated sapodilla (40 °C, 50 °Brix, 165 min) presented microbiological standards that met those established by Resolution RDC No. 12 of 2 January 2001, of the ‘Agência Nacional de Vigilância Sanitária’ [40] (Table 6), thus demonstrating good manufacturing practices.

### 3.3. Centesimal Composition and Physical–Chemical Characteristics

Table 7 shows the centesimal composition of the sapodilla in natura and osmotically dehydrated. Sapodilla in natura has a similar profile to most fruits, exhibiting a high content of moisture and carbohydrates, and low contents of proteins and lipids. The contents of carbohydrates and proteins were similar (20.40 g/100 g and 0.38 g/100 g, respectively) to those values previously reported by Gonsalves [39] (20 g/100 g and 0.4 g/100 g, respectively) However, the total caloric value was higher (93.70 g/100 g) than that found by Gonsalves [39] (83 g/110 g). All parameters of the centesimal composition differed significantly (*p* < 0.05) between sapodilla in natura and dehydrated, except for ash content (Table 7). In addition, the ash content of the sapodilla in natura (0.48%) was similar for the value found by Uekane et al. [11] (0.5%).

The contents of proteins, lipids, carbohydrates, fibers and TCV of the dehydrated fruit was significantly higher (*p* < 0.05) than the in natura fruit, a fact explained by the dehydration of the fruit, consequently elevating the concentrations of these nutrients. This dehydration allowed significant loss of water from the fruit (53.23%) (Table 7). The water activity (Aw), soluble solids and pH data are shown in Table 8. It is possible to observe that the soluble solid contents of the sapodilla dehydrated increased in relation to sapodilla in natura.

This fact was explained by the concentration of sapodilla’s natural sugars and the incorporation of sucrose during the process. The pH remained practically the same after the drying process. As for Aw, the dehydrated fruit is characterized as an intermediate moisture fruit. In another study, several parameters for fresh and lyophilized sapodilla pulp values were analyzed: 15.67 °Brix and 65.50 °Brix were observed, respectively. The pH values of fresh sapodilla (5.55) and lyophilized fruit (5.58) were also similar [20]. After osmotic dehydration, the sapodilla was submitted to drying in a greenhouse, in a humidity of 24.34%. This value is within the limits established by Brazilian legislation [40].

### 3.4. Structural Analysis of Osmotically Dehydrated Fruit

**Figure 6:**
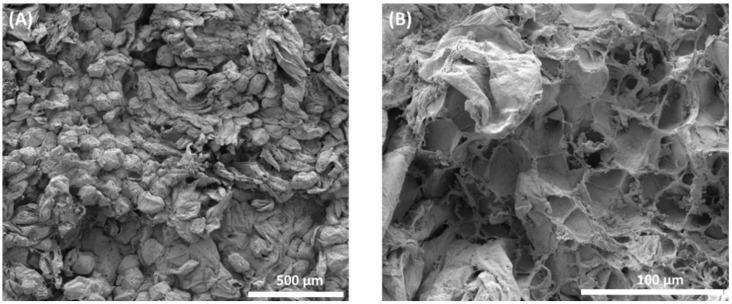
Microstructural analysis of osmotically dehydrated sapodilla. (**A**) General microscopic aspect of dehydrated sapodilla showing disordered cells; (**B**) areas of cell wall rupture.

Over the last few years, there have been changes in the field of osmotic processing research and, in addition to the evaluation of macroscopic aspects, it has been sought to observe the process on a microscopic scale, because view of the anatomy and physiology of plant tissue and its cells is useful for understanding mass transfer phenomena [35]. Figure 6A,B shows the cellular structures of sapodilla subjected to the osmotic dehydration process, followed by drying. In Figure 6A, it can be observed that the cells are disordered, there is no uniformity in size due to the wrinkling caused by the loss of water during osmotic dehydration and the turgor of the cells is reduced.

Plant tissues submitted to the osmotic process tended to have cells with smaller volumes and sizes than in natura tissue [41]. Sapodilla cells in natura had a mean size of 123.7 μm × 105 μm, and osmotically dehydrated sapodilla showed cells with a mean size of 121.9 μm × 97.55 μm.

Figure 6B shows the rupture of the cell walls in a certain area, emphasizing that most of the cells remained with their structure intact. Some factors may contribute to the rupture of cell walls, such as lower cell wall resistance due to pectin solubilization, high osmotic pressure which generates changes in the distribution of pressure gradients in the tissue and causes the cells that are in direct contact with the osmotic solution to lose water and release the turgor pressure, and cell wrinkling occurs [41,42]. With the loss of water, denaturation of the protein occurs, resulting in damaged membranes. This type of damage leads to a decreased cell wall and cell membrane, and possibly cell death [39].

The microstructural differences induced by the application of osmotic dehydration in autumn olives are also shown by the images. The analysis carried out on the osmotically dehydrated fruit showed that the microstructure of the fruit became smoother, with some cracks on the surface. This observation denoted that the osmotic dehydration process affected the overall mass transport properties of the tissue. In addition, the cells shrank and some collapsed after water loss [43].

Similar structural changes were found after the dehydration of pumpkin cylinders and guava halves. In the in natura vegetables, the cells were swollen, and after dehydration, there was non-homogeneous shrinkage of these cells. In the cellular tissue of the guava, there was extensive cellular plasmolysis and the cells appeared to be deformed and wrinkled [44,45].

## 4. Conclusions

This study reports the development of an effective process for the osmotic dehydration of sapodilla fruit. The results show that moisture loss and solid gains were influenced by the temperature and concentration of the osmotic solution. In addition, the osmotic solution concentration and immersion time had significant influence on the Dehydration Efficiency Index. Based on moisture loss, two samples were subjected to sensorial analysis. Sample 9 (submitted to a sucrose solution of 50 °Brix, under operation temperature of 40 °C by 165 min) obtained the highest scores of acceptability and purchase intention. It also showed compliance with the microbiological standards and current Brazilian legislation for dehydrated fruits. Finally, the osmotic treatment generated turgor reduction and the rupture of sapodilla cell walls. Taken together, the data obtained indicate that the studied process is a good alternative for the conservation of sapodilla.

## Figures and Tables

**Table 1:** Coded and decoded levels of independent variables.

Variables	−α	−1	0	1	+α
**Temperature (°C)**	30	34	40	46	50
**Concentration of the osmotic solution (°Brix)**	40	44	50	56	60
**Time of immersion (minutes)**	90	120	165	210	240

**Table 2:** Effects of independent variables on the ML, SG and DEI of sapodilla.

Assay	Temperature (°C)	%Sucrose	Immersion Time (Minutes)	ML	SG	DEI
**1**	34	44	120	23.8	9.56	2.49
**2**	46	44	120	28.23	8.51	3.32
**3**	34	56	120	30.77	14.40	2.14
**4**	46	56	120	34.9	9.10	3.83
**5**	34	44	210	23.08	5.68	4.06
**6**	46	44	210	33.19	15.20	2.18
**7**	34	56	210	34.03	7.80	4.36
**8**	46	56	210	45.04	12.10	3.72
**9**	40	50	165	39.27	9.90	3.97
**10**	40	50	165	38.93	10.10	3.85
**11**	40	50	165	38.25	9.80	3.9
**12**	30	50	165	28.8	9.27	3.11
**13**	50	50	165	38.19	12.17	3.14
**14**	40	40	165	25.61	10.80	2.37
**15**	40	60	165	38.16	11.46	3.33
**16**	40	50	90	31.7	9.80	3.23
**17**	40	50	240	36.81	10.70	3.44

ML, moisture loss; SG, solid gain; DEI, Dehydration Efficiency Index.

**Table 3:** Regression coefficients of the quadratic equation for ML, SG and DEI.

	ML	*p*	SG	*p*	DEI	*p*
**β_0_**	−262.09	0.005	1.936	0.769	−28.638	0.006
**β_1_**	−4.334	0.010	−0.134	0.414	0.531	0.009
**β_11_**	−0.061	0.005	0.005	0.063	−0.006	0.006
**β_2_**	7.582	0.004	0.978	0.021	0.537	0.011
**β_22_**	−0.077	0.003	0.009	0.020	−0.009	0.003
**β_3_**	−0.091	0.212	−0.225	0.004	0.084	0.005
**β_33_**	−0.001	0.007	0.00003	0.901	−0.0001	0.014
**β_12_**	0.002	0.722	−0.033	0.002	0.007	0.006
**β _13_**	0.006	0.013	0.009	0.0004	−0.002	0.001
**β_23_**	0.004	0.025	−0.003	0.004	0.0008	0.010
**R^2^**	0.982		0.979		0.947	

ML, moisture loss (%); SG, solid gain (%); DEI, Dehydration Efficiency Index. The regression coefficients represent: β_0_ = average; β_1_ = the linear term of temperature; β_11_ = the quadratic term of temperature; β_2_ = the linear term of osmotic solution concentration; β_22_ = the quadratic term of osmotic solution concentration; β_3_ = the linear term of immersion time; β_33_ = the quadratic term of osmotic solution concentration; β_12_ = term of the interaction between temperature and concentration of the osmotic solution; β_13_ = term of the interaction between temperature and concentration of the osmotic solution; β_23_ = term of the interaction between osmotic solution concentration and immersion time; R^2^ = determination coefficient.

**Table 4:** Mean acceptance scores for dehydrated sapodilla (assays 7 and 9).

Assay	Aroma	Color	Taste	Texture	Global Quality
**7**	6.50 ± 1.25 ^a^	6.30 ± 1.47 ^a^	6.64 ± 1.34 ^a^	6.42 ± 1.54 ^a^	6.44 ± 1.31 ^a^
**9**	6.72 ± 1.41 ^a^	6.58 ± 1.44 ^a^	6.78 ± 1.58 ^a^	6.82 ± 1.69 ^a^	6.76 ± 1.39 ^a^

In each column, means followed by the same superscript letter (^a^) do not differ significantly at the 5% level of significance by the Student’s *t*-test.

**Table 5:** Purchase intention for dehydrated sapodilla (assays 7 and 9).

Assay	I Would Certainly Not Buy	Maybe I Would Not Buy	Maybe I Would Buy/Maybe I Would Not Buy	Maybe I Would Buy	I Would Certainly Buy
**7**	2%	14%	30%	**32%**	22%
**9**	4%	4%	**34%**	26%	**32%**

**Table 6:** Microbiological analysis of osmotically dehydrated sapodilla (assay 9).

Microbiological Analysis	Result	Legislation
**Coliforms 45 °C/g**	<0.3 MPN	Max. 10^2^/g
***Salmonella* sp./25g**	Absence in 25 g	Absence in 25 g

MPN: Most probable number.

**Table 7:** Centesimal composition of in natura and osmotically dehydrated sapodilla.

Parameter *	Sapodilla	
	In Natura	Dehydrated Osmotically + Drying (Assay 9)
**Humidity (g/100 g)**	77.57 ± 0.23 ^a^	24.34 ± 0.20 ^b^
**Ashes (g/100 g)**	0.48 ± 0.09 ^a^	0.45 ± 0.05 ^a^
**Proteins (g/100 g)**	0.38 ± 0.02 ^b^	0.73 ± 0.02 ^a^
**Lipids (g/100 g)**	1.18 ± 0.006 ^b^	2.53 ± 0.08 ^a^
**Carbohydrates (g/100 g)**	20.40 ± 0.16 ^b^	71.95 ± 0.14 ^a^
**Fibers (g/100 g)**	1.06 ± 0.13 ^b^	2.68 ± 0.38 ^a^
**TCV (kcal)**	93.70 ± 0.61 ^b^	313.51 ± 0.81 ^a^

TCV, total caloric value. * Averages of 3 determinations. In each line, means followed by the same superscript letters (^a,b^) do not differ significantly at the 5% level of significance by the Student’s *t*-test.

**Table 8:** Physicochemical characteristics of in natura and osmotically dehydrated sapodilla.

Physicochemical characteristics *	Sapodilla	
	In Natura	Dehydrated Osmotically + Drying (Assay 9)
**Water activity (Aw)**	0.985 ± 0.001 ^a^	0.803 ± 0.017 ^b^
**Total soluble solids (°Brix)**	13.67 ± 0.58 ^b^	19.67 ± 2.08 ^a^
**pH**	4.75 ± 0.05 ^a^	4.71 ± 0.32 ^a^

TCV, total caloric value. * Averages of 3 determinations. In each line, means followed by the same letters (^a,b^) do not differ significantly at the 5% level of significance by the Student’s *t*-test.

## Data Availability

The data presented in this study are available on request from the corresponding author.
